# Human-robot interaction in sustainable hospitality: how robot type shapes customer emotions, green perceptions, and service loyalty

**DOI:** 10.3389/frobt.2026.1845884

**Published:** 2026-06-17

**Authors:** Kok Meng Ng, Aliya Bayakhmetova

**Affiliations:** 1 Asia Pacific University of Technology and Innovation, Kuala Lumpur, Malaysia; 2 Almaty Management University, Almaty, Kazakhstan

**Keywords:** service robots, human-robot interaction, perceived sustainability, customer satisfaction, revisit intention

## Abstract

The increasing adoption of service robots in hospitality has transformed service delivery and created new opportunities for enhancing operational efficiency and sustainable service practices. However, limited research has examined how different types of service robots influence customer emotions and how these emotional responses shape perceptions of sustainability and post-consumption behaviour. Drawing on the Stimulus–Organism–Response (S-O-R) framework and Human–Robot Interaction (HRI) theory, this study investigates the relationships among robot type, emotional responses, perceived sustainability, customer satisfaction and revisit intention in hotel settings. A quantitative cross-sectional survey was conducted among 238 hotel guests in Malaysia who had directly interacted with either high-interaction robots (e.g. front-desk or concierge robots) or low-interaction robots (e.g. delivery robots). Data were analysed using Partial Least Squares Structural Equation Modelling (PLS-SEM). The findings reveal that high-interaction robots significantly increase both positive and negative emotions. Positive emotions positively influence perceived sustainability and customer satisfaction, whereas negative emotions weaken these evaluations. Perceived sustainability significantly enhances customer satisfaction and revisit intention, while customer satisfaction emerges as the strongest predictor of revisit intention. Emotional responses also mediate the relationship between robot type and customer outcomes. This study extends HRI and sustainable hospitality literature by demonstrating that customer evaluations of robot-enabled services are shaped by emotional appraisal and sustainability perception rather than technological functionality alone. The findings provide practical insights for hospitality managers seeking to design emotionally engaging and sustainability-oriented robot-enabled service experiences.

## Introduction

The hospitality industry is undergoing rapid transformation as artificial intelligence (AI) and automation become increasingly embedded in service operations. Among the most visible manifestations of this transformation is the growing adoption of service robots in hotels. Service robots, defined as AI-enabled agents capable of autonomously or semi-autonomously interacting with customers and performing service-related tasks, are now used in a wide range of hospitality functions, including front desk reception, concierge assistance, housekeeping support and in-room delivery (Wirtz et al., 2018; Wirtz et al., 2024). Their increasing presence reflects the industry’s broader shift toward technology-enabled service systems designed to improve operational efficiency, reduce labour dependency and enhance service consistency.

The adoption of service robots has accelerated in response to both structural and environmental pressures. Persistent labour shortages, rising operational costs and the growing demand for standardized service delivery have encouraged hospitality firms to invest in automation. These developments were further intensified by the COVID-19 pandemic, which increased consumer demand for contactless, hygienic and low-touch service encounters, making service robots particularly attractive in hotel environments (Mukherjee et al., 2021; Hao, 2021). From an operational perspective, service robots offer clear advantages: they can perform repetitive tasks with high precision, reduce service errors and operate continuously without fatigue, thereby improving efficiency and service reliability (Qiu et al., 2024). As a result, many hotels increasingly regard service robots as strategic assets capable of enhancing both productivity and customer experience.

Despite these advantages, the integration of robots into hospitality remains theoretically and managerially complex because hospitality is fundamentally a human-centred service domain. Unlike purely transactional service environments, hospitality is characterized by emotional engagement, interpersonal warmth and personalized interaction. Customers often evaluate service quality not only on functional outcomes but also on relational cues such as empathy, responsiveness and authenticity. While robots may perform efficiently, they remain limited in their ability to reproduce the emotional nuance and social intelligence typically associated with human employees (Belanche et al., 2021; Song et al., 2023). This creates an important tension in hospitality service design: robots may improve efficiency, but they may also alter the emotional quality of the service experience (Dan et al., 2021; Ghazali et al., 2025; Song et al., 2025).

This tension has made human-robot interaction (HRI) an increasingly important area of inquiry in hospitality research. Existing studies show that customer responses to service robots are often emotionally mixed. On one hand, robot interactions may generate enjoyment, novelty and curiosity, particularly when customers perceive robots as innovative or entertaining. On the other hand, the same interactions may evoke anxiety, discomfort or social awkwardness, especially when robots are perceived as impersonal, unfamiliar or unable to respond with human-like sensitivity (Belanche et al., 2021; Lee and Song, 2023). These findings suggest that customer responses to service robots are not uniformly positive or negative, but emotionally ambivalent. Yet much of the existing literature has tended to examine these emotional outcomes in isolation, often emphasizing either positive engagement or negative resistance rather than acknowledging their simultaneous coexistence.

A second limitation in the literature concerns the treatment of service robots as functionally homogeneous. Prior studies often discuss service robots as a single category, despite substantial differences in how robots interact with customers and deliver service. In practice, service robots vary considerably in their level of interaction intensity. High-interaction robots, such as front desk or concierge robots, are designed to engage directly with customers through communication, information exchange and social interaction. By contrast, low-interaction robots, such as delivery robots, perform primarily functional tasks with minimal interpersonal engagement. This distinction is theoretically important because the intensity and social nature of robot interaction are likely to shape customer expectations, emotional responses and service evaluations in different ways (Mukherjee et al., 2021; Christensen et al., 2025). High-interaction robots may generate stronger emotional reactions due to their greater social presence, whereas low-interaction robots may be evaluated primarily on efficiency and convenience (Apraiz et al., 2023). However, empirical research rarely differentiates robot types in a systematic way, limiting understanding of how specific service robot roles shape customer experience.

A third and increasingly important issue concerns the relationship between service robots and sustainability. Sustainability has become a strategic priority in hospitality as firms face growing pressure to reduce environmental impact, improve operational efficiency and align with broader societal expectations regarding responsible business practices. These concerns are closely aligned with the United Nations Sustainable Development Goals (SDGs), particularly SDG 9 (Industry, Innovation and Infrastructure), SDG 12 (Responsible Consumption and Production) and SDG 13 (Climate Action), which emphasize the importance of technological innovation, sustainable operations and resource efficiency in service industries. In this context, service robots are increasingly positioned as part of sustainable service innovation because they may contribute to energy optimization, waste reduction and more efficient resource allocation (Khan and Fatma, 2023). For example, delivery robots may reduce unnecessary movement and improve operational routing, while AI-enabled systems may support more efficient allocation of labour and energy across hotel operations.

However, the sustainability value of service robots should not be assumed uncritically (Qiu et al., 2024). Although robots may improve operational efficiency, their actual environmental contribution is not always straightforward. Service robots require energy-intensive hardware, digital infrastructure and maintenance systems, all of which may generate environmental costs. Their sustainability value therefore depends not only on technical efficiency but also on how such technologies are deployed, integrated and evaluated. More importantly, sustainability in service settings is not determined solely by objective operational outcomes. Customer perceptions of sustainability often matter as much as, or more than, technical performance in shaping service evaluations and behavioural intentions (Hao, 2021; Lazim et al., 2025). Even when service robots generate operational efficiencies, customers may not necessarily interpret their presence as environmentally responsible (Christensen et al., 2025; Ghazali et al., 2025). This distinction between actual and perceived sustainability remains underexplored in the service robot literature.

The limited research that addresses sustainability in hospitality technology has also tended to conceptualise sustainability perception as a rational and cognitive judgment. Yet customer evaluations of service experiences are not purely cognitive; they are shaped by affective responses that influence how service encounters are interpreted and remembered. Emotions play a central role in shaping customer judgments, particularly in technology-mediated interactions where uncertainty, novelty and social interpretation are heightened (Jang et al., 2015). Positive emotions such as enjoyment and curiosity may lead customers to interpret robot-enabled services more favourably, including as more innovative and environmentally responsible. Conversely, negative emotions such as anxiety and discomfort may weaken the perceived value of automation and reduce the extent to which customers associate such services with sustainability. This suggests that perceived sustainability may be shaped not only by what service robots do, but by how customers feel when interacting with them.

Taken together, these gaps indicate that the current literature offers only a partial understanding of how service robots influence customer evaluations in hospitality. Existing studies have largely examined robot adoption through narrow lenses of technology acceptance, functional service quality or isolated emotional responses, while paying limited attention to how robot type, emotional ambivalence and sustainability perception interact in shaping customer outcomes. This fragmented perspective limits both theoretical development and practical guidance. To address this gap, the present study develops and tests an integrated framework grounded in the Stimulus-Organism-Response (S-O-R) framework and HRI theory. Specifically, the study conceptualises robot type as the service stimulus, emotional responses as the internal organismic state and perceived sustainability, customer satisfaction and revisit intention as downstream evaluative and behavioural responses.

By integrating these dimensions into a single framework, this study offers a more comprehensive explanation of how customers interpret robot-enabled service experiences in hospitality. It contributes to the literature in three important ways. First, it advances HRI research by showing that service robots evoke both positive and negative emotions simultaneously rather than in isolation. Second, it extends sustainable hospitality research by demonstrating that perceived sustainability is shaped not only by operational logic but also by emotional appraisal. Third, it provides a more integrated explanation of how robot-enabled service experiences influence customer satisfaction and loyalty in hospitality settings. In doing so, the study offers both theoretical and practical insight into the role of service robots in shaping the future of sustainable hospitality.

## Literature review

### Theoretical foundation: S-O-R framework and human-robot interaction (HRI)

This study is grounded in the Stimulus-Organism-Response (S-O-R) framework and Human-Robot Interaction (HRI) theory, which together provide a comprehensive explanation of how customers respond to service robots in hospitality contexts. The S-O-R framework posits that external stimuli influence individuals’ internal states, which subsequently shape behavioural responses. In this study, robot type is conceptualised as the stimulus, emotional responses as the organism, and perceived sustainability, customer satisfaction, and revisit intention as the responses.

While the S-O-R model has been widely applied in consumer behaviour research, its application to robot-enabled services remains limited. Existing studies often focus on technology acceptance variables such as perceived usefulness and ease of use, overlooking the emotional mechanisms through which technological stimuli influence behavioural outcomes. This represents a critical gap, particularly in service contexts where emotional experience plays a central role. Complementing the S-O-R framework, HRI theory emphasizes the importance of social presence, interaction quality, and anthropomorphic characteristics in shaping user responses to robots. Research suggests that robots capable of social interaction can enhance engagement but may also trigger discomfort when expectations are not met. This duality highlights the need to examine both positive and negative emotional responses within a unified framework. By integrating S-O-R and HRI perspectives, this study provides a theoretically grounded explanation of how different types of service robots influence customer emotions and subsequent evaluations.

### Service robots in hospitality: opportunities and tensions

The adoption of service robots in the hospitality industry has accelerated in recent years, driven by broader advancements in artificial intelligence (AI) and automation technologies. Service robots are increasingly deployed to perform a variety of functions, ranging from front desk operations and concierge services to housekeeping and delivery tasks. These robots are designed to enhance operational efficiency, reduce labour dependency, and provide consistent service performance, particularly in standardized service encounters (Wirtz et al., 2024). As hotels face rising labour costs and increasing pressure to improve productivity, the adoption of robotic technologies has become a strategic priority.

Many empirical studies suggest that service robots are particularly effective in executing routine and repetitive tasks that require precision and reliability. Their ability to operate continuously without fatigue allows hospitality firms to maintain service consistency while optimizing resource utilization (Chen et al., 2021). In addition, the growing demand for contactless services, especially following the COVID-19 pandemic, has further accelerated the adoption of robots as they minimize physical interaction and enhance perceived safety (Hao, 2021; Qiu et al., 2024). More recent research also highlights their role in reshaping workforce structures and service delivery models (Elmohandes et al., 2026).

However, the literature presents contradictory findings regarding customer responses to service robots. While some studies report enhanced satisfaction and novelty-driven engagement, others indicate reduced perceived warmth and emotional connection (Tussyadiah, 2020; Belanche et al., 2021; Stock-Homburg, 2022). This tension reflects a fundamental challenge in hospitality: balancing efficiency with emotional service quality. According to Wang (2025), customers often seek personalized attention, warmth, and emotional engagement, which remain challenging for robots to replicate. As a result, while robots may enhance efficiency, their impact on overall customer experience remains contingent upon how customers perceive and interact with them.

Furthermore, existing research has largely focused on technology acceptance models (e.g., UTAUT), which emphasize cognitive evaluations but often neglect emotional and experiential factors. For instance, recent work using meta-UTAUT frameworks (Marghany et al., 2025) demonstrates the importance of perceived usefulness and social influence but does not fully capture the emotional complexity of human–robot interaction. These limitations suggest the need to move beyond purely cognitive models and incorporate affective and experiential dimensions, particularly in high-contact service environments.

### Robot type in service encounters: beyond a homogeneous view

An important consideration in understanding customer responses to service robots is the distinction between different types of robots used in hospitality settings. Existing research indicates that robots are not a homogeneous technological category; rather, they differ significantly in terms of functionality, interaction level, and social presence (Qiu et al., 2024). In particular, the distinction between high-interaction and low-interaction robots has emerged as a critical factor influencing customer perceptions and evaluations.

High-interaction robots, such as those deployed at hotel front desks or concierge stations, are designed to engage directly with customers through verbal and non-verbal communication. These robots often simulate social interaction by providing information, answering queries, and sometimes displaying human-like expressions or gestures. Due to their interactive nature, customers are more likely to evaluate these robots based on social and emotional criteria, such as friendliness, warmth, and responsiveness (Ghazali et al., 2025). These robots enhance social presence, which can increase engagement and perceived innovativeness. However, research also suggests that higher levels of anthropomorphism may lead to discomfort when robots fail to meet human-like expectations, a phenomenon often associated with the “uncanny valley” effect (Terrah et al., 2026).

In contrast, low-interaction robots are primarily designed for functional efficiency and task execution. Examples include delivery robots that transport items to guest rooms or robotic cleaners that operate with minimal human involvement. These robots typically require limited customer interaction and are evaluated more on their performance attributes, such as speed, accuracy, and convenience (Tiruwa et al., 2025; de Oliveira Santini et al., 2025). Because of their utilitarian nature, low-interaction robots may generate fewer emotional responses but can still influence overall service evaluation through perceived efficiency.

The distinction between robot types is important because it suggests that customer expectations and experiences vary depending on the level of interaction. High-interaction robots may trigger stronger emotional reactions due to their social presence, whereas low-interaction robots may be perceived as tools that enhance operational efficiency. Understanding these differences is essential for developing a more nuanced perspective on human-robot interaction in hospitality.

### Emotional responses in human-robot interaction

Emotional responses play an important role in influencing customer evaluations of service experiences, and this is particularly evident in the context of human-robot interaction. When customers interact with service robots, they often experience a range of emotions that can influence their attitudes, satisfaction, and behavioural intentions. These emotional responses can be broadly categorized into positive and negative dimensions (Tussyadiah, 2020).

Positive emotions such as enjoyment, excitement, and curiosity are often linked to the novelty and innovation of interactions with robots. Customers may view robot services as entertaining or futuristic, enhancing their overall experience and increasing their willingness to engage with the technology (Tiruwa et al., 2025; de Oliveira Santini et al., 2025). These positive emotional responses have been shown to contribute to higher satisfaction levels and favourable behavioural outcomes, including a greater likelihood of repeat use and positive word-of-mouth.

At the same time, human-robot interaction can also generate negative emotions. Customers may feel anxiety, discomfort, or even fear when interacting with robots, particularly if the technology appears unfamiliar or lacks human-like warmth. Concerns about reliability, privacy, and loss of human touch can further exacerbate these negative emotions (Tussyadiah, 2020; Ghazali et al., 2025). The presence of such negative emotional responses can hinder technology acceptance and reduce overall satisfaction.

Importantly, existing studies tend to examine either positive or negative emotions in isolation, resulting in an incomplete understanding of customer responses. However, emerging evidence suggests that these emotional responses can coexist, particularly in interactions involving high levels of social presence.

From a theoretical perspective, this duality can be explained through affective evaluation theory, which posits that individuals form judgments based on both positive and negative emotional cues. This suggests that customer evaluations of robot services are not purely rational but are shaped by complex emotional processes.

### Perceived sustainability: moving beyond assumptions

Sustainability has become an increasingly important factor in consumer decision-making, especially in the hospitality industry. Perceived sustainability refers to customers’ assessment of a firm’s environmental responsibility and its efforts to reduce negative environmental impacts (Khan and Fatma, 2023). With growing environmental awareness, customers are more inclined to favor businesses that show a genuine commitment to sustainable practices.

In the context of hospitality, sustainability initiatives often include energy conservation, waste reduction, and the use of environmentally friendly technologies (Shih et al., 2024). Service robots are frequently positioned as part of these initiatives due to their potential to improve operational efficiency and optimize resource utilization. For example, robots can reduce unnecessary energy consumption by performing tasks more efficiently and minimizing human error (Soori et al., 2023). Additionally, the integration of AI technologies can support smart systems that enhance overall environmental performance (Huang and Rust, 2021a).

However, while the technical benefits of service robots are well documented, the extent to which customers perceive these technologies as sustainable remains unclear. According to Kim et al. (2015), customer perception is a critical factor because it influences how sustainability initiatives are evaluated and whether they translate into positive behavioural outcomes. If customers do not associate robot usage with environmental benefits, the strategic value of such technologies in promoting sustainability may be limited.

Furthermore, perceived sustainability is not formed in isolation but may be influenced by other factors, including emotional responses. Positive emotions may enhance customers’ perceptions of a firm’s environmental responsibility, while negative emotions may undermine these perceptions. Despite its importance, the relationship between emotional responses and perceived sustainability in robot-enabled services has received limited empirical attention, indicating a need for further investigation.

### Customer satisfaction and revisit intention

Customer satisfaction is widely acknowledged as a critical driver of behavioural intentions in service settings. It reflects the degree to which a service experience meets or surpasses customer expectations and plays a central role in fostering loyalty and revisit intention (Singh et al., 2023). In the hospitality industry, satisfied customers are more likely to return, recommend the service to others, and build long-term relationships with the service provider (Tuncer et al., 2021).

The introduction of service robots adds a new dimension to the satisfaction construct. Studies have shown that the quality of robot-delivered services can significantly influence customer satisfaction, particularly when the technology meets expectations in terms of efficiency, reliability, and ease of use (Tuncer et al., 2021; Huang and Rust, 2021b). Positive experiences with robot services can enhance overall satisfaction, while negative experiences may lead to dissatisfaction and reduced loyalty.

Revisit intention, a critical behavioural outcome, is strongly associated with customer satisfaction. Guests who are satisfied with their service experience are more likely to return to the same hotel and continue using its offerings. In robot-enabled hospitality, both emotional responses and perceived sustainability can influence satisfaction, which in turn impacts revisit intention. This indicates that satisfaction acts as a mediating factor through which different influences shape customer loyalty.

### Hypotheses development

Drawing on the S-O-R framework and HRI theory, this study proposes a series of hypotheses linking robot type, emotional responses, sustainability perception, and behavioural outcomes.

The first set of hypotheses focuses on the relationship between robot type and emotional responses. Prior research suggests that the level of interaction between customers and service robots plays a critical role in shaping emotional reactions. High-interaction robots, such as those used at the front desk, possess greater social presence and engage customers in direct communication, thereby intensifying emotional experiences (Ghazali et al., 2025). These interactions can evoke positive emotions such as enjoyment, curiosity, and excitement due to the novelty and perceived innovativeness of the technology (Paulose and Shakeel, 2022; Tuncer et al., 2021). At the same time, the increased social exposure associated with high-interaction robots may also trigger negative emotions, including anxiety or discomfort, particularly when customers perceive the interaction as unnatural or lacking human warmth (Belanche et al., 2021). In contrast, low-interaction robots typically perform functional tasks with minimal engagement, resulting in weaker emotional responses. Hence, robot type, as a stimulus, is expected to influence emotional responses due to differences in interaction intensity. High-interaction robots enhance social presence and engagement, leading to stronger positive emotions. However, they may also increase negative emotions due to uncertainty and unmet expectations.Based on this reasoning, the following hypotheses are proposed:

H1 High-interaction robots positively influence positive emotional responses.

H2 High-interaction robots positively influence negative emotional responses.

The second set of hypotheses explores the impact of emotional responses on perceived sustainability. Emotions play a key role in shaping how customers interpret and assess their service experiences, including their perceptions of a firm’s environmental responsibility. Positive emotions can enhance cognitive evaluations by creating favorable impressions and strengthening trust in the service provider (Jang et al., 2015; Farolfi et al., 2021). When customers feel enjoyment or satisfaction during interactions with robots, they are more likely to view the technology as beneficial and aligned with sustainable practices. In contrast, negative emotions such as anxiety or discomfort may foster scepticism and diminish the perceived credibility of sustainability claims (Ghazali et al., 2025). Therefore, emotional responses are expected to shape how customers perceive the environmental impact of robot-enabled services. Based on this reasoning, the following hypotheses are proposed:

H3 Positive emotional responses positively influence perceived sustainability.

H4 Negative emotional responses negatively influence perceived sustainability.

The third set of hypotheses examines the relationship between perceived sustainability and customer outcomes. Perceptions of sustainability are widely recognized as key drivers of customer attitudes and behavioural intentions, particularly in environmentally sensitive sectors like hospitality (Jang et al., 2015; Paulose and Shakeel, 2022). When customers view a hotel as environmentally responsible, they are more likely to form positive evaluations of the service, resulting in higher levels of satisfaction. Additionally, sustainability perception can enhance a firm’s reputation and strengthen customer trust, which in turn increases the likelihood of revisit intention (Lazim et al., 2025). In the context of robot-enabled services, if customers associate the use of robots with environmental benefits such as improved energy efficiency and reduced waste they are more likely to respond positively to the service experience. Based on this, the following hypotheses are proposed:

H5 Perceived sustainability positively influences customer satisfaction.

H6 Perceived sustainability positively influences revisit intention.

The fourth set of hypotheses focuses on the direct relationship between emotional responses and customer satisfaction. Emotions are central to service evaluation, as they capture the immediate affective reactions of customers during service encounters (Tussyadiah, 2020; Singh et al., 2023; Soori et al., 2023). Positive emotions, such as enjoyment and excitement, are expected to boost satisfaction by creating enjoyable and engaging experiences, whereas negative emotions, such as anxiety and discomfort, are likely to lower satisfaction by undermining perceived service quality. In the context of human-robot interaction, these emotional responses are particularly salient because they reflect customers’ reactions to a relatively novel and unfamiliar service format (Tussyadiah, 2020). Accordingly, the following hypotheses are proposed:

H7 Positive emotional responses positively influence customer satisfaction.

H8 Negative emotional responses negatively influence customer satisfaction.

Finally, the relationship between customer satisfaction and revisit intention is well established in the consumer behaviour literature (Utami et al., 2023). Satisfaction represents a cumulative evaluation of the service experience and serves as a key predictor of future behavioural intentions (Dan et al., 2021; Huang and Rust, 2021a; Apraiz et al., 2023). Customers who are satisfied with robot-enabled services are more likely to revisit the same hotel and continue using its offerings. Conversely, dissatisfaction may prompt switching behaviour and generate negative word-of-mouth. Recognizing the critical role of satisfaction in fostering customer loyalty, the following hypothesis is proposed:

H9 Customer satisfaction positively influences revisit intention.

Overall, the proposed hypotheses reflect a comprehensive framework in which robot type influences emotional responses, which subsequently shape perceived sustainability and customer satisfaction, ultimately leading to revisit intention. This integrated approach provides a deeper understanding of how technological and psychological factors interact to influence customer behaviour in sustainable hospitality contexts.

To synthesize the current state of knowledge and clarify the positioning of the present study, Table 1 summarizes key studies on service robots and sustainable hospitality. The review shows that prior research has largely focused on technology acceptance, operational efficiency and customer attitudes toward service robots. While these studies provide valuable insights, three important gaps remain evident. First, robot types are rarely differentiated based on interaction intensity. Second, emotional responses are often examined in isolation rather than as coexisting positive and negative reactions. Third, the relationship between robot-enabled services and perceived sustainability remains underexplored, particularly in relation to customer satisfaction and loyalty. These limitations provide the basis for the conceptual model developed in this study.

**TABLE 1:** Summarizes key studies on service robots and sustainable hospitality.

Author(s)	Context	Focus	Key findings	Limitation/Research gap
Wirtz et al. (2024)	Service industries	Service robots in frontline service/services delivery	Established foundational understanding of service robots as AI-enabled service agents that improve efficiency and consistency	Primarily conceptual; limited empirical insight into customer emotional responses
Belanche et al. (2021)	Hospitality/tourism	Customer acceptance of service robots	Customers respond positively to robot usefulness and novelty, but may also experience discomfort and reduced perceived warmth	Focuses mainly on acceptance; does not examine sustainability perceptions
Mukherjee et al. (2021)	Hospitality	Contactless service during COVID-19	Service robots increased perceived safety and hygiene in hotel environments	Context-specific to pandemic conditions; limited long-term behavioural implications
Tussyadiah (2020)	Tourism and hospitality	Emotional responses to robot interaction	Robot interaction generates both enjoyment and anxiety, suggesting emotionally mixed responses	Positive and negative emotions examined, but not linked to sustainability or loyalty outcomes
Huang and Rust (2021a)	Service management	AI and automation in service	AI improves service efficiency, standardization and operational reliability	Emphasizes operational performance; limited focus on customer emotional evaluation
Ghazali et al. (2025)	Hospitality	Robot interaction intensity	High-contact robots generate stronger emotional and social responses than low-contact robots	Does not examine sustainability perceptions or post-consumption behaviour
Hao, F. (2021)	Hospitality	Acceptance of technology	Perceived of technologies interaction and its influences on satisfaction and revisit intention in hospitality settings	Focuses technologies in hospitality including the usage of AI or robot-enabled services
Shih et al. (2024)	Smart hospitality	AI and sustainable operations	AI-enabled systems can improve resource efficiency and support sustainable hotel operations	Focuses on operational sustainability, not customer perception of sustainability
Marghany et al. (2025)	Hotels	Guest acceptance of service robots	Robot acceptance is shaped by performance expectancy, trust and social influence	Focuses on adoption intention rather than emotional ambivalence or sustainability
Elmohandes et al. (2026)	Restaurants	Technology and workforce transformation	Service automation improves efficiency but requires careful human–technology role balancing	Focuses on workforce implications rather than customer emotional or sustainability outcomes
Terrah et al. (2026)	Restaurants	Anthropomorphic robot design	Anthropomorphic features improve attractiveness and engagement in HRI	Focuses on design attractiveness; sustainability implications remain unexplored

### Conceptual framework

Based on the theoretical relationships discussed, this study develops a conceptual framework that illustrates the interplay between robot type (stimulus), emotional responses (organism), perceived sustainability, and customer outcomes (response). Robot type serves as the primary antecedent, influencing both positive and negative emotional responses. These emotional responses, in turn, affect perceived sustainability, which subsequently influences customer satisfaction and revisit intention. In addition, emotional responses are proposed to have a direct impact on satisfaction, reflecting their central role in shaping service evaluation.

**FIGURE 1:**
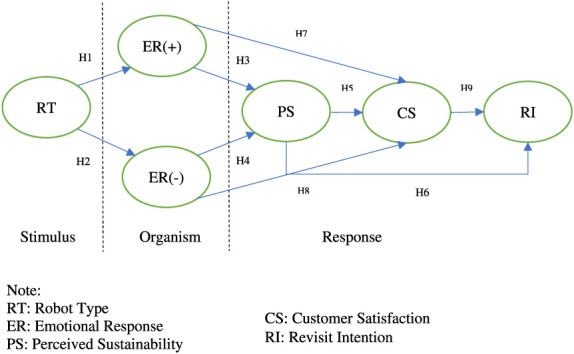
Conceptual Framework (Source: Author’s own work).

The framework (as shown in Figure 1) reflects a sequential process in which technological characteristics influence psychological responses, which then shape cognitive evaluations and behavioural intentions. By integrating these elements into a single model, the study provides a comprehensive perspective on human-robot interaction in sustainable hospitality.

## Methodology

### Research design

This study adopted a quantitative, cross-sectional design to examine how robot type influences customer emotional responses, perceived sustainability, satisfaction and revisit intention in hotel service settings. A quantitative approach was appropriate because the study seeks to test a theoretically specified model grounded in the Stimulus-Organism-Response (S-O-R) framework and Human-Robot Interaction (HRI) theory. In line with this logic, robot type represents the external stimulus, emotional responses capture the internal organismic state and perceived sustainability, customer satisfaction and revisit intention constitute the response outcomes. A cross-sectional design was selected because it allows the capture of customers’ evaluations immediately following a naturally occurring service encounter involving a hotel service robot. This design is widely used in hospitality and consumer research when the objective is to examine theoretically grounded relationships among latent constructs in real service environments (Lia and Vassilis, 2015; Creswell and Creswell, 2018; Ghanad, 2023). Although cross-sectional data do not permit causal inference over time, the design is appropriate for theory testing in context-specific service encounters and is consistent with prior HRI research examining customer evaluations of robot-mediated services (Belanche et al., 2021; Ghazali et al., 2025). The study follows a deductive approach. Hypotheses were developed from prior theory and empirical evidence and subsequently tested using survey data collected from customers with actual service robot experience. This approach is suitable for validating the proposed conceptual model and examining the mechanisms through which service robots influence customer perceptions and behavioural outcomes.

### Research context

The empirical setting for this study was the hotel sector, where service robots have become increasingly visible in front-office and delivery operations. Hotels provide an appropriate context for examining HRI because hospitality services are characterized by high customer contact, strong emotional expectations and frequent service evaluation. These characteristics make hotels particularly suitable for investigating how customers respond to service robots beyond functional performance. The study was conducted in Malaysia, an emerging hospitality market where digital transformation in tourism and accommodation has accelerated in recent years. The Malaysian context is theoretically relevant for two reasons. First, the adoption of service technologies in Malaysian hotels has increased in response to labour constraints, operational efficiency pressures and growing investment in smart tourism infrastructure. Second, Malaysia represents an emerging market context where technological novelty remains salient and customer expectations toward service automation are still evolving. This provides a meaningful setting for examining emotional and evaluative responses to service robots. The Malaysian setting also extends the geographical scope of existing HRI research, which has predominantly focused on technologically mature economies such as Japan, China, South Korea and the United States. By examining robot-enabled service experiences in an emerging market, this study contributes to the broader generalisability of HRI and hospitality research.

### Sampling and data collection

The target population for this study comprised hotel guests who had directly interacted with a service robot during their stay. Because the study investigates post-interaction emotional and evaluative responses, only respondents with actual experience using a hotel service robot were considered eligible to participate. This inclusion criterion was necessary to ensure that respondents could provide informed evaluations based on real service encounters rather than hypothetical assumptions. To recruit eligible respondents, this study employed a non-probability intercept sampling approach, combining purposive screening with convenience-based recruitment. This approach is more appropriate than a purely purposive sampling label because respondents were recruited based on accessibility in hotel settings, while eligibility was determined through a screening criterion requiring prior interaction with a service robot. In methodological terms, this reflects a convenience sampling strategy with purposive qualification rather than strict purposive sampling in its classical sense (Bryman and Bell, 2015).

This distinction is important. Purposive sampling is typically used when participants are deliberately selected for possessing highly specific expertise or characteristics central to theory development. While respondents in this study did need direct robot interaction experience, they were not selected through researcher judgment based on specialized characteristics. Rather, respondents were approached based on practical accessibility in participating hotels and subsequently screened for eligibility. As such, the sampling process aligns more closely with convenience sampling, which is widely used in hospitality and consumer research where respondents are recruited in naturally occurring service environments (Bryman and Bell, 2015). This approach is particularly appropriate when the objective is to examine consumer evaluations immediately following a specific service experience.

The use of convenience-based intercept sampling is also consistent with prior hospitality and HRI research, where respondents are commonly recruited at service locations following actual interaction with technology-enabled services. This method is appropriate for studies examining real-time customer perceptions because it allows researchers to capture context-specific evaluations while minimizing recall bias. In the present study, hotel guests were approached in hotel lobbies, check-out areas and service zones shortly after interacting with a service robot and invited to complete a structured questionnaire. To ensure construct relevance, respondents were first screened with a qualifying question asking whether they had interacted with a hotel service robot during their stay. Only those answering affirmatively were invited to participate. Before completing the questionnaire, respondents were also provided with brief descriptions and visual examples of high-interaction robots (e.g., front desk or concierge robots) and low-interaction robots (e.g., delivery robots) to ensure consistent interpretation of robot type.

Data were collected using a structured self-administered questionnaire distributed immediately after the service encounter or at the point of hotel checkout. Collecting responses close to the interaction reduced retrospective distortion and improved the accuracy of emotional reporting, which is especially important in HRI research where affective reactions are highly time-sensitive. However, this timing may also heighten transient affective responses, particularly novelty-related excitement or short-term discomfort. This issue is acknowledged as a limitation and discussed further in Chapter 5. A total of 256 questionnaires were collected. After excluding incomplete responses and cases that failed the screening criterion, 238 usable responses were retained for analysis. This sample size exceeds the minimum threshold recommended for PLS-SEM and is sufficient for estimating the proposed structural model with adequate statistical power (Hair and Alamer, 2022).

Although convenience-based intercept sampling limits statistical generalisability, it is appropriate for theory-testing research conducted in natural service settings where access to respondents is context-dependent. Consistent with Bryman and Bell (2015), the objective of this study is not statistical representativeness of the broader population, but analytical generalisation to theory. The sampling strategy is therefore appropriate given the study’s aim of examining theoretically grounded relationships among customers with direct experience of robot-enabled hotel services.

### Measures and instrument development

The survey instrument was developed using established multi-item scales adapted from prior literature and refined for the hospitality robot context. This approach supports content validity while ensuring alignment with existing empirical work. All constructs were measured using seven-point Likert scales ranging from 1 (“strongly disagree”) to 7 (“strongly agree”), consistent with standard practice in consumer and hospitality research. Robot type was operationalised as a categorical predictor distinguishing between high-interaction and low-interaction robots. High-interaction robots referred to customer-facing robots used in front desk or concierge functions, where direct communication and social interaction were central to the service encounter. Low-interaction robots referred to delivery robots used primarily for functional tasks with limited interpersonal engagement. To reduce ambiguity and ensure consistent interpretation, respondents were provided with brief descriptions and visual examples of both robot types before completing the questionnaire. This classification is consistent with prior studies that emphasize the varying levels of interaction in human-robot encounters (Dan et al., 2021; Qiu et al., 2024).

Emotional responses were conceptualised as two distinct affective dimensions: positive emotions and negative emotions. Positive emotions captured favourable affective reactions such as enjoyment and curiosity, whereas negative emotions captured anxiety and discomfort. These measures were adapted from Belanche et al. (2021) and revised to reflect robot-mediated hotel service encounters. Perceived sustainability was defined as the customer’s evaluation of the extent to which robot-enabled services support environmentally responsible hotel operations. This includes perceptions of operational efficiency, reduced resource waste and environmental responsibility. Measurement items were adapted from Shih et al. (2024), with modifications to reflect the role of service robots in supporting sustainable hospitality operations. Customer satisfaction was assessed as an overall evaluation of the service experience, reflecting the degree to which customer expectations were met or exceeded. The measurement items encompass both cognitive and affective dimensions of the service encounter and are based on the widely accepted conceptualization of satisfaction in consumer behaviour research (Singh et al., 2023). Finally, revisit intention was assessed through respondents’ stated likelihood of returning to the hotel and recommending it to others. These behavioural intention measures are commonly used in hospitality research as indicators of customer loyalty and future engagement. The items were adapted from established scales in prior studies (Shih et al., 2024), ensuring consistency with existing research on customer retention and word-of-mouth behaviour. The instrument underwent a content validity review by three hospitality and HRI experts to ensure clarity, relevance, and alignment with research objectives. A pilot study involving 30 hotel customers was conducted to assess the reliability and clarity of the questionnaire. The results demonstrated satisfactory internal consistency, with Cronbach’s alpha values exceeding 0.70 for all constructs (Hair et al., 2021). Minor adjustments were made to the wording of certain items to enhance clarity and comprehension.

### Analytical procedure

Data analysis was conducted using Partial Least Squares Structural Equation Modelling (PLS-SEM) in three sequential stages. First, descriptive analysis was performed to summarize respondent demographics and service robot interaction characteristics. This provided contextual insight into the sample profile and service encounter conditions. Second, the measurement model was assessed to establish construct reliability and validity. Internal consistency reliability was evaluated using Cronbach’s alpha and composite reliability. Convergent validity was assessed through outer loadings and average variance extracted (AVE). Discriminant validity was evaluated using both the Fornell–Larcker criterion and the heterotrait–monotrait ratio (HTMT), following current best practice. Third, the structural model was assessed to test the hypothesized relationships. Path coefficients, significance levels, coefficients of determination (R^2^) and effect sizes (f^2^) were examined to evaluate both statistical and practical significance. To estimate the significance and stability of the structural paths, bootstrapping with 5,000 resamples was performed.

In addition to direct effects, mediation analysis was conducted to examine whether emotional responses mediated the effects of robot type on perceived sustainability and customer satisfaction. Indirect effects were assessed using bootstrapped confidence intervals, allowing for more precise evaluation of the underlying explanatory mechanisms. PLS-SEM was selected for three reasons. First, it is well suited to theory extension and prediction-oriented research. Second, it is appropriate for analysing complex models with multiple mediating relationships. Third, it performs well with moderate sample sizes and does not impose strict distributional assumptions, making it suitable for the present study.

### Common method bias and ethical considerations

Because the study relied on self-reported data collected from a single source, procedural and statistical remedies were employed to reduce the risk of common method bias. Procedurally, respondents were assured anonymity and confidentiality, questionnaire items were carefully worded to reduce ambiguity and social desirability bias and predictor and criterion constructs were separated in the survey layout to reduce response pattern effects. Statistically, Harman’s single-factor test was conducted as an initial diagnostic. The results indicated that no single factor accounted for the majority of the variance, suggesting that common method bias was unlikely to pose a serious threat. In addition, full collinearity variance inflation factor (VIF) values were examined and remained below the recommended threshold, providing further evidence that common method bias was not a substantial concern.

This study involved human participants and was conducted in accordance with established ethical standards for social science research. Participation was voluntary and informed consent was obtained prior to data collection. Respondents were informed of the purpose of the study and assured that their responses would remain anonymous and confidential. No personally identifiable information was collected and participants were informed that they could withdraw from the study at any time without consequence. The study adhered to institutional ethical guidelines for research involving human participants.

The methodology aligns with prior research on human-robot interaction and sustainable hospitality (Belanche et al., 2021; Jang et al., 2015; Shih et al., 2024). By combining purposive sampling, validated measurement instruments, and SEM analysis, the study captures both the affective and cognitive dimensions of customer responses, while generating robust empirical evidence with practical and theoretical implications. Applying SEM to survey data from hotel customers provides a rigorous framework for testing the effects of robot type on emotional responses, perceived sustainability, satisfaction, and revisit intention.

## Results analysis

### Respondent profile

A total of 238 valid responses (return rate of 79.3% out of 300) were obtained and used for analysis after the data screening. The demographic profile provides important contextual insight into the nature of respondents engaging with service robots. The demographic characteristics of the respondents are summarized in Table 2.

**TABLE 2:** Respondent demographic profile (N = 238).

Variable	Category	Frequency	Percentage (%)
Gender	Male	112	47.1
	Female	126	52.9
Age	18–25	78	32.8
	26–35	102	42.9
	36–45	38	16.0
	46 and above	20	8.3
Experience type	High-interaction	121	50.8
	Low-interaction	117	49.2

The gender distribution appears relatively balanced, suggesting that the findings are not skewed toward a particular gender group. More importantly, the age distribution indicates that a large proportion of respondents fall within the 18-35 age range, which is consistent with prior research indicating that younger consumers are more receptive to technological innovations and AI-driven services.

The nearly equal distribution between high-interaction and low-interaction robot experiences is particularly significant. It strengthens the validity of comparative insights regarding robot type, ensuring that observed differences in emotional responses are not driven by sample imbalance. This balance also allows for a more reliable interpretation of how varying levels of human–robot interaction influence customer perceptions.

### Descriptive statistics

Table 3 reports the descriptive statistics for the study constructs. Positive emotions recorded the highest mean score, indicating that respondents generally perceived robot interactions as enjoyable and engaging. Negative emotions recorded a lower mean, suggesting that discomfort and anxiety were present but less dominant. Perceived sustainability and customer satisfaction were moderately high, indicating generally favourable evaluations of robot-enabled service experiences.

These results suggest that customers generally viewed robot-enabled service encounters positively, although negative emotional responses remained present and non-trivial.

**TABLE 3:** Descriptive statistics.

Construct	Mean	SD
Positive emotions	5.42	0.89
Negative emotions	3.18	1.11
Perceived sustainability	5.11	0.94
Customer satisfaction	5.27	0.88
Revisit intention	5.34	0.91

### Measurement model assessment

The measurement model was assessed to evaluate reliability and validity of the constructs, including emotional responses (ER), perceived sustainability (PS), customer satisfaction (CS), and revisit intention (RI).

### Reliability analysis

Internal consistency reliability was assessed using Cronbach’s alpha and composite reliability (CR). As shown in Table 4, all values exceeded the recommended threshold of 0.70 (Hair et al., 2021).

**TABLE 4:** Reliability and convergent validity.

Construct	Items	Loading range	Cronbach’s alpha	Composite reliability (CR)	AVE
Positive emotions	4	0.781–0.884	0.88	0.91	0.72
Negative emotions	4	0.744–0.852	0.85	0.89	0.68
Perceived sustainability	4	0.768–0.887	0.87	0.90	0.69
Customer satisfaction	4	0.801–0.911	0.90	0.93	0.76
Revisit intention	3	0.816–0.915	0.89	0.92	0.74

The results indicate that all constructs exhibit high internal consistency reliability. Cronbach’s alpha values range from 0.85 to 0.90, exceeding the recommended threshold of 0.70. This indicates that the items within each construct are measuring the same underlying concept consistently. Composite reliability (CR), which is considered a more robust measure in SEM, further confirms this observation, with all values above 0.89. This suggests that the constructs are stable and free from excessive measurement error. Convergent validity is assessed through the Average Variance Extracted (AVE). All AVE values exceed 0.50, indicating that each construct explains more than half of the variance of its indicators. Notably, customer satisfaction, CS (AVE = 0.76) and revisit intention, RI (AVE = 0.74) show particularly strong convergence, suggesting that these outcome variables are measured with a high degree of precision.

Overall, these findings confirm that the measurement model meets the required standards for reliability and convergent validity, allowing for meaningful structural analysis.

### Discriminant validity

Discriminant validity was assessed using both the Fornell–Larcker criterion and the HTMT ratio. As shown in Table 5, the HTMT values are all below the conservative threshold of 0.90, indicating that there is no significant overlap between constructs. A closer examination reveals that the relationship between customer satisfaction (CS) and revisit intention (RI) (HTMT = 0.78) is relatively strong compared to other pairs. This is theoretically expected, as satisfaction is a direct antecedent of revisit intention. However, the value remains below the threshold, confirming that the two constructs, while related, are not redundant. Similarly, the moderate HTMT values between emotions and sustainability suggest that while emotional responses influence sustainability perception, they remain conceptually distinct constructs. This reinforces the validity of modeling emotions as antecedents rather than components of sustainability perception.

**TABLE 5:** HTMT results.

Constructs	PE	NE	PS	CS	RI
Positive emotions (ER+)	—				
Negative emotions (ER-)	0.42	—			
Perceived sustainability	0.61	0.55	—		
Customer satisfaction	0.68	0.49	0.70	—	
Revisit intention	0.65	0.45	0.72	0.78	—

Table 6 shows the square root of AVE for each construct exceeded its inter-construct correlations, satisfying the Fornell–Larcker criterion.

**TABLE 6:** Discriminant validity (fornell–larcker criterion).

Construct	1	2	3	4	5
1. Positive emotions	0.840				
2. Negative emotions	−0.284	0.817			
3. Perceived sustainability	0.521	−0.337	0.828		
4. Customer satisfaction	0.612	−0.401	0.576	0.857	
5. Revisit intention	0.483	−0.289	0.542	0.689	0.882

### Structural model assessment

The structural model was evaluated using path coefficients (β), t-values, and significance levels (p-values) obtained through bootstrapping (5,000 resamples).

### Coefficient of determination (R^2^)

The explanatory power of the model is presented in Table 7.

**TABLE 7:** R^2^ values.

Endogenous construct	R^2^ value	Interpretation
Positive emotions	0.32	Moderate
Negative emotions	0.28	Moderate
Perceived sustainability	0.41	Moderate
Customer satisfaction	0.56	Substantial
Revisit intention	0.63	Substantial

The R^2^ values provide insight into the predictive power of the model. The model explains 63% of the variance in revisit intention (RI), which is considered substantial in behavioural research. This indicates that the combination of satisfaction and sustainability perceptions plays a strong role in shaping customer loyalty. Customer satisfaction (CS) also shows a relatively high R^2^ value of 0.56, suggesting that it is well explained by emotional responses (ER) and perceived sustainability (PS). This reinforces the central role of satisfaction as a mediating construct linking emotional and cognitive evaluations to behavioural outcomes. In contrast, the R^2^ values for positive (0.32) and negative emotions (0.28) are moderate. This implies that while robot type is an important predictor, other unexamined factors (e.g., personality traits, prior experience with technology) may also influence emotional responses. This opens avenues for future research.

### Collinearity assessment

Variance inflation factor (VIF) values in Table 8 were below the recommended threshold of 3.3, indicating that multicollinearity was not a concern in the structural model.

These values indicate that collinearity does not bias the structural estimates.

**TABLE 8:** Collinearity assessment (VIF).

Path predictor	VIF
Robot type	1.000
Positive emotions	1.384
Negative emotions	1.384
Perceived sustainability	1.512
Customer satisfaction	1.476

### Hypothesis testing

The results of hypothesis testing are summarized in Table 9.

**TABLE 9:** Structural model results (hypothesis testing).

Hypothesis	Path	β	t-value	p-value	f^2^	Result
H1	Robot type → positive emotions	0.57	9.12	<0.001	0.271	Supported
H2	Robot type → negative emotions	0.33	5.48	<0.001	0.061	Supported
H3	Positive emotions → sustainability	0.46	7.21	<0.001	0.183	Supported
H4	Negative emotions → sustainability	−0.29	4.89	<0.001	0.052	Supported
H5	Sustainability → satisfaction	0.52	8.03	<0.001	0.096	Supported
H6	Sustainability → revisit intention	0.41	6.77	<0.001	0.074	Supported
H7	Positive emotions → satisfaction	0.34	5.92	<0.001	0.171	Supported
H8	Negative emotions → satisfaction	−0.21	3.88	<0.001	0.061	Supported
H9	Satisfaction → revisit intention	0.49	9.45	<0.001	0.312	Supported

The hypothesis testing results provide strong empirical support for the conceptual model. The effect of robot type (RT) on positive emotions (ER+) (β = 0.57) is particularly strong, indicating that high-interaction robots significantly enhance customer enjoyment and engagement. At the same time, the positive relationship with negative emotions (ER-) (β = 0.33) suggests that increased interaction also introduces elements of discomfort or uncertainty, reflecting the dual nature of human-robot interaction. The influence of emotions on perceived sustainability (PS) is especially noteworthy. Positive emotions significantly enhance sustainability perceptions, suggesting that customers interpret technological efficiency more favourably when their experience is enjoyable. Conversely, negative emotions weaken sustainability perceptions, indicating that discomfort may overshadow perceived environmental benefits.

Perceived sustainability shows a strong impact on both satisfaction (CS) (β = 0.52) and revisit intention (RI) (β = 0.41), highlighting its strategic importance. This finding suggests that sustainability is not merely a background attribute but an active driver of customer evaluation and loyalty. The direct effects of emotions on satisfaction further reinforce their importance. Positive emotions (ER+) enhance satisfaction (CS), while negative emotions reduce it, demonstrating that emotional experiences play a critical role beyond cognitive evaluations. Finally, customer satisfaction (CS) exerts a strong influence on revisit intention (RI) (β = 0.49), confirming its role as a key determinant of behavioural outcomes. This aligns with well-established theories in consumer behaviour and hospitality research.

### Mediation analysis

To examine the indirect mechanisms underlying customer responses, mediation analysis was conducted using bootstrapped indirect effects. The results show that emotional responses significantly mediated the relationship between robot type and downstream evaluations.

As shown in Table 10, positive emotions significantly mediated the effect of robot type on perceived sustainability and customer satisfaction. This suggests that high-interaction robots enhance favourable evaluations primarily by increasing enjoyment and curiosity. Negative emotions also exerted significant indirect effects, but in the opposite direction, weakening sustainability perception and satisfaction.

**TABLE 10:** Mediation analysis (indirect effects).

Indirect path	Beta	t-value	p-value	Decision
Robot type → positive emotions → perceived sustainability	0.193	4.982	0.000	Supported
Robot type → negative emotions → perceived sustainability	−0.042	2.631	0.009	Supported
Robot type → positive emotions → customer satisfaction	0.181	4.641	0.000	Supported
Robot type → negative emotions → customer satisfaction	−0.047	2.418	0.016	Supported

These findings provide stronger evidence that emotional responses serve as the primary psychological mechanism through which robot type influences customer perceptions and service evaluations.

## Discussion of key findings

This study examined how robot type shapes customer emotional responses and how these emotional reactions subsequently influence perceived sustainability, customer satisfaction and revisit intention in hotel service settings. The findings provide strong support for the proposed model and offer several important theoretical insights into human-robot interaction (HRI) in hospitality.

First, the results show that robot type plays a foundational role in shaping customer responses. High-interaction robots significantly increased both positive and negative emotions, confirming that robot-mediated service encounters generate emotionally ambivalent responses rather than uniformly positive or negative evaluations. This finding extends prior HRI research by demonstrating that socially interactive robots simultaneously function as sources of engagement and discomfort. While previous studies have often examined customer enjoyment or anxiety in isolation, the present findings show that these emotional reactions coexist and are jointly activated in high-contact robot encounters (Belanche et al., 2021; Ghazali et al., 2025). From a broader perspective, these findings align with Sustainable Development Goal (SDG) 9: Industry, Innovation and Infrastructure, as they demonstrate how advanced technologies such as service robots can enhance service innovation and transform customer experiences within the hospitality sector.

The simultaneous increase in positive and negative emotions can be understood through the combined logic of HRI theory and the S-O-R framework. High-interaction robots increase social presence, novelty and perceived innovativeness, which stimulate curiosity and enjoyment. At the same time, greater interaction intensity also raises cognitive and emotional demands, increasing the likelihood of discomfort, uncertainty and social awkwardness. This supports the view that customer responses to service robots are shaped not only by functional performance but also by the tension between technological novelty and unmet social expectations. This duality is also consistent with the “uncanny valley” perspective, which suggests that technologies perceived as socially intelligent may enhance engagement while simultaneously generating discomfort when they fail to fully replicate human interaction. From a sustainability standpoint, this suggests that technological innovation alone is insufficient; its success depends on user acceptance and emotional comfort, which are critical for achieving long-term sustainable adoption in line with SDG 9.

Second, the findings demonstrate that emotional responses serve as a critical explanatory mechanism linking robot interaction to downstream evaluations. Positive emotions significantly enhanced perceived sustainability and customer satisfaction, whereas negative emotions weakened both outcomes. This finding is theoretically important because it shows that customers do not evaluate robot-enabled services solely on functional or environmental grounds. Rather, sustainability perception is partly affective in nature and shaped by how customers feel during the interaction.

This extends prior sustainability research, which has largely conceptualised perceived sustainability as a rational or cognitive evaluation of environmental responsibility. The present findings suggest that sustainability judgments are also emotionally constructed. Customers who experience enjoyment and curiosity appear more likely to interpret robot-enabled efficiency as environmentally beneficial, whereas customers who experience discomfort or anxiety appear less likely to perceive the same service as sustainable. This demonstrates that sustainability perception is not merely an outcome of operational performance, but also a psychologically mediated judgment. This insight extends prior sustainability research by demonstrating that sustainability perception is not purely cognitive but also emotionally constructed. Importantly, this finding supports SDG 12: Responsible Consumption and Production, as it highlights that consumers’ willingness to support sustainable practices depends not only on operational improvements but also on how such practices are experienced and perceived.

Third, perceived sustainability emerged as a significant predictor of both customer satisfaction and revisit intention, confirming that sustainability has become a meaningful evaluative criterion in hospitality consumption. This finding reinforces the growing view that customers increasingly incorporate environmental responsibility into their service evaluations and post-consumption behavioural intentions. Importantly, the results suggest that sustainability in robot-enabled hospitality is not simply a background operational issue, but a visible and behaviourally relevant component of customer experience (Shih et al., 2024). In addition, by linking sustainability perception to behavioural outcomes, the study indirectly contributes to SDG 13: Climate Action, as it demonstrates how environmentally oriented innovations can influence consumer behaviour and encourage more sustainable consumption patterns.

Fourth, customer satisfaction remained the strongest direct predictor of revisit intention, reinforcing its central role in hospitality behaviour. However, the present findings also show that satisfaction is shaped by a broader set of antecedents than traditionally assumed. In robot-enabled service settings, satisfaction is not driven solely by efficiency or service quality, but also by emotional experience and sustainability perception. This expands conventional hospitality models by showing that customer loyalty in technology-mediated service environments is shaped by a more complex interplay of cognitive, affective and ethical evaluations theories (Tuncer et al., 2021; Utami et al., 2023).

Taken together, the findings demonstrate that service robots influence customer behaviour through a layered evaluative process in which technological characteristics shape emotional reactions, emotional reactions shape sustainability perceptions and these evaluations ultimately influence satisfaction and loyalty. This provides a more complete explanation of how robot-enabled service experiences are interpreted by customers and why they produce divergent behavioural outcomes.

### Theoretical contributions

This study makes four specific theoretical contributions to the literature on human-robot interaction (HRI), hospitality and sustainable service research.

First, the study advances HRI theory by demonstrating that customer responses to service robots are emotionally ambivalent rather than unidimensional. Much of the existing HRI literature has tended to examine positive responses such as enjoyment, novelty and engagement or negative responses such as anxiety and discomfort as separate and independent outcomes. This fragmented treatment has limited theoretical understanding of how customers actually experience robot-mediated services. The present study shows that high-interaction robots simultaneously intensify both positive and negative emotional responses, suggesting that emotional ambivalence is a structurally embedded feature of socially interactive robot encounters rather than a peripheral reaction. This extends HRI theory by moving beyond binary assumptions of either acceptance or resistance and offering a more realistic account of customer emotional processing in robot-enabled service environments.

Second, the study extends the Stimulus-Organism-Response (S-O-R) framework by demonstrating that emotional responses do not merely mediate behavioural outcomes, but also shape evaluative judgments about sustainability. In most S-O-R applications, emotions are positioned as internal organismic states that explain downstream outcomes such as satisfaction or behavioural intention. The present study extends this logic by showing that emotions also influence how customers interpret the environmental meaning of a service encounter. Specifically, the findings demonstrate that positive emotions increase perceived sustainability, whereas negative emotions weaken it. This contribution is theoretically important because it repositions perceived sustainability as an affectively mediated judgment rather than a purely rational evaluation of operational efficiency. In doing so, the study broadens the explanatory scope of S-O-R and introduces a more psychologically grounded understanding of sustainability perception in service contexts, thereby supporting SDG 12.

Third, the study contributes to sustainable hospitality research by challenging the dominant assumption that sustainability evaluations are formed primarily through rational assessments of environmental performance. Existing sustainable hospitality literature has largely conceptualised sustainability as a cognitive judgment based on visible green practices, operational efficiency or environmental responsibility. This study shows that sustainability perception is also emotionally constructed. Customers do not interpret robot-enabled services as sustainable simply because they appear technologically efficient; rather, they are more likely to assign sustainability value when the interaction is emotionally positive. This finding contributes to sustainable hospitality theory by demonstrating that sustainability perception is not only operationally derived, but also affectively filtered through the service experience.

Fourth, the study develops and empirically validates an integrated framework linking robot type, emotional responses, perceived sustainability, customer satisfaction and revisit intention. Existing research has largely examined these constructs in isolation, often focusing separately on technology acceptance, emotional reaction or sustainable consumption. As a result, prior models have offered only partial explanations of how service robots influence customer behaviour. By integrating these constructs into a single explanatory model, the present study offers a more comprehensive account of how technological service stimuli are translated into behavioural outcomes. This integrated framework advances theory by clarifying the psychological and evaluative mechanisms through which service robots influence post-consumption behaviour in hospitality settings.

Finally, the findings reinforce the importance of mediating mechanisms, particularly the role of emotions and sustainability perception in translating technological features into behavioural outcomes which aligned with previous studies by de Oliveira Santini et al. (2025). This contributes to a deeper theoretical understanding of how service robots can support not only operational efficiency but also broader sustainability goals, including those related to SDG 13.

Taken together, these contributions move the literature beyond narrow technology acceptance explanations by demonstrating that customer responses to service robots are shaped by a more complex interplay of technological stimuli, emotional appraisal and sustainability interpretation. In doing so, the study offers a more theoretically complete explanation of customer behaviour in robot-enabled hospitality services.

### Practical contributions

This study also offers several specific and actionable contributions for hospitality managers, service designers and technology decision-makers seeking to implement service robots in ways that enhance customer experience and long-term service value.

First, the findings show that service robot implementation should be managed as an experiential design issue rather than solely as an operational efficiency initiative. Hospitality firms often adopt service robots to improve speed, consistency and labour efficiency. However, the results show that customer responses depend not only on what robots do, but also on how customers feel when interacting with them. High-interaction robots may improve engagement, but they also increase emotional discomfort if the interaction feels unclear, unnatural or socially awkward. This suggests that service robot implementation should be guided by customer experience design principles rather than technological functionality alone. For practitioners, this means that high-interaction robots should be designed with emotional usability in mind. Hotels should prioritize clear dialogue structures, intuitive interfaces, predictable responses and socially appropriate interaction cues. Rather than maximizing anthropomorphic sophistication, managers should focus on reducing ambiguity and cognitive strain during customer interaction. In practical terms, simpler and more predictable robot behaviour may generate stronger customer acceptance than highly human-like but socially imperfect interaction.

Second, the findings suggest that robot deployment strategies should be differentiated by service role. The results show that robot type matters because interaction intensity shapes emotional response. This means that hotels should not deploy all robots in the same way or evaluate them using the same performance criteria. High-interaction robots are more suitable for engagement-oriented tasks such as information provision, check-in assistance and wayfinding, where novelty and interaction can enhance customer experience. Low-interaction robots are better suited to functional and efficiency-driven tasks such as item delivery, room service support and logistics, where customers prioritise speed and convenience over social interaction. This role-based deployment strategy allows hotels to align robot design with customer expectations and reduce emotional mismatch (Tiruwa et al., 2025).

Third, the study demonstrates that sustainability benefits must be actively communicated to customers rather than assumed. Hotels may adopt service robots for operational sustainability reasons, such as reducing labour inefficiencies, optimizing energy use or improving resource allocation. However, the findings show that customers do not automatically perceive robot-enabled services as environmentally responsible. Their sustainability judgments are shaped by emotional experience and service framing. This means that hotels must make the environmental value of service robots visible and understandable to customers. Practically, this can be achieved by embedding sustainability cues into the service encounter. Hotels can communicate robot-related sustainability benefits through digital interfaces, in-room messaging, check-in prompts or service signage that explains how robots contribute to reduced waste, lower energy use or more efficient service delivery. Making these sustainability benefits explicit can improve both customer perceptions and downstream loyalty outcomes.

Fourth, the findings highlight the importance of customer onboarding in reducing negative emotional responses. Anxiety and discomfort in robot interaction are often driven by unfamiliarity, uncertainty and lack of control. This means that even well-designed service robots may generate resistance if customers do not understand how to interact with them. Hotels should therefore treat onboarding as part of the service experience. Simple orientation cues, first-use instructions, visual prompts and immediate access to human assistance can reduce uncertainty and improve emotional comfort, especially for first-time users.

Finally, the study supports the use of hybrid service models in which robots and human employees perform complementary rather than competing roles. The findings show that while robots are effective in enhancing efficiency and novelty, they are less capable of delivering empathy, reassurance and adaptive emotional support. Hotels should therefore avoid full substitution models and instead adopt hybrid service systems where robots handle routine, repetitive and standardized tasks while human employees manage emotionally complex, high-ambiguity or recovery-related interactions. This approach allows firms to preserve operational efficiency without sacrificing the relational qualities that remain central to hospitality service.

In short, these contributions provide more actionable guidance for hospitality managers by showing that successful robot deployment depends not only on technological capability, but on the strategic alignment between robot design, emotional experience, service role and sustainability communication.

## Limitations and conclusion

This study provides important insights into how robot type shapes customer emotions, perceived sustainability and post-consumption behaviour in hotel service settings, but several limitations should be acknowledged when interpreting the findings.

First, the study employed a cross-sectional design, which limits the ability to establish temporal causality among the constructs. Although the hypothesized relationships are theoretically grounded and empirically supported, customer emotions and evaluations may evolve over repeated interactions with service robots. Emotional novelty, in particular, may diminish over time as customers become more familiar with robot-enabled services. Future research should therefore adopt longitudinal designs to examine how emotional responses, sustainability perceptions and behavioural intentions change across repeated encounters.

Second, the study relied on convenience-based intercept sampling within Malaysian hotels, which may limit the generalisability of the findings across cultural and service contexts. Although Malaysia offers a relevant and theoretically meaningful emerging market setting, customer expectations toward automation may differ in technologically mature economies or in service environments with different cultural norms regarding technology and social interaction. Future studies should replicate the model across countries and hospitality segments to test its external validity and cultural robustness.

Third, the study used self-reported measures collected immediately after service encounters. This approach improved recall accuracy and allowed emotional responses to be captured close to the interaction itself, but it may also have amplified transient affective reactions, particularly novelty-related excitement or short-term discomfort. Future studies may strengthen validity by combining self-reported evaluations with observational, experimental or behavioural data, such as actual revisit behaviour, interaction duration or facial/emotional response tracking.

Fourth, although the proposed model explains substantial variance in customer satisfaction and revisit intention, it does not capture all relevant mechanisms that may shape customer responses to service robots. Constructs such as trust, anthropomorphism, perceived intelligence, technology readiness and service familiarity may provide additional explanatory power and should be incorporated in future research to extend the model.

Fifth, the study focused on two broad robot categories based on interaction intensity, namely, high-interaction and low-interaction robots. While this distinction is theoretically useful, it does not account for more granular robot characteristics such as voice design, emotional expressiveness, anthropomorphic appearance or adaptive intelligence. Future research should examine how specific design features influence emotional and behavioural outcomes in greater depth.

Despite these limitations, the study makes several important contributions to the literature on human–robot interaction, sustainable hospitality and customer behaviour. The findings demonstrate that customer evaluations of service robots are shaped not only by technological functionality, but by a layered psychological process in which robot type influences emotional responses, emotional responses shape sustainability perceptions and these evaluations ultimately influence satisfaction and revisit intention. This study therefore moves beyond narrow technology acceptance explanations by showing that customer responses to service robots are simultaneously emotional, evaluative and behavioural.

The study contributes to theory by extending HRI and S-O-R perspectives through a more integrated explanation of how customers interpret robot-enabled service encounters. In particular, it demonstrates that emotional ambivalence is a central feature of high-interaction robot encounters and that perceived sustainability is not solely a rational judgment, but one shaped by emotional appraisal. These insights provide a more complete theoretical explanation of how service robots influence customer outcomes in hospitality settings.

The study also contributes to practice by showing that successful robot deployment depends not only on technological capability, but on the strategic alignment between robot design, emotional usability and sustainability communication. Hotels seeking to implement service robots effectively must therefore manage robots not merely as operational tools, but as experiential service touchpoints that shape customer perceptions and loyalty.

In conclusion, the successful integration of service robots in hospitality depends on more than automation efficiency alone. As service robots become increasingly embedded in hotel operations, their long-term value will depend on how well they align with customer emotions, service expectations and perceptions of sustainable value. Understanding this broader evaluative process is essential for designing robot-enabled hospitality experiences that are not only technologically effective, but also emotionally acceptable, commercially viable and operationally sustainable.

## Data Availability

The raw data supporting the conclusions of this article will be made available by the authors, without undue reservation.
